# Biosurfactant Production in Sub-Oxic Conditions Detected in Hydrocarbon-Degrading Isolates from Marine and Estuarine Sediments

**DOI:** 10.3390/ijerph17051746

**Published:** 2020-03-07

**Authors:** Patrícia M. Domingues, Vanessa Oliveira, Luísa Seuanes Serafim, Newton C. M. Gomes, Ângela Cunha

**Affiliations:** 1 Department of Chemistry and CICECO, University of Aveiro, 3810-193 Aveiro, Portugal; 2 Department of Biology and CESAM, University of Aveiro, 3810-193 Aveiro, Portugal

**Keywords:** deep-sea, estuary, anaerobic, atomized oil assay, petroleum hydrocarbons, anoxic

## Abstract

Hydrocarbon bioremediation in anoxic sediment layers is still challenging not only because it involves metabolic pathways with lower energy yields but also because the production of biosurfactants that contribute to the dispersion of the pollutant is limited by oxygen availability. This work aims at screening populations of culturable hydrocarbonoclastic and biosurfactant (BSF) producing bacteria from deep sub-seafloor sediments (mud volcanos from Gulf of Cadiz) and estuarine sub-surface sediments (Ria de Aveiro) for strains with potential to operate in sub-oxic conditions. Isolates were retrieved from anaerobic selective cultures in which crude oil was provided as sole carbon source and different supplements were provided as electron acceptors. Twelve representative isolates were obtained from selective cultures with deep-sea and estuary sediments, six from each. These were identified by sequencing of 16S rRNA gene fragments belonging to *Pseudomonas*, *Bacillus*, *Ochrobactrum*, *Brevundimonas*, *Psychrobacter*, *Staphylococcus*, *Marinobacter* and *Curtobacterium* genera. BSF production by the isolates was tested by atomized oil assay, surface tension measurement and determination of the emulsification index. All isolates were able to produce BSFs under aerobic and anaerobic conditions, except for isolate DS27 which only produced BSF under aerobic conditions. These isolates presented potential to be applied in bioremediation or microbial enhanced oil recovery strategies under conditions of oxygen limitation. For the first time, members of *Ochrobactrum*, *Brevundimonas*, *Psychrobacter*, *Staphylococcus*, *Marinobacter* and *Curtobacterium* genera are described as anaerobic producers of BSFs.

## 1. Introduction

Biosurfactants (BSFs) are amphiphilic molecules that alter the surface and interfacial tensions, promoting the dispersion of one phase into the other [1]. BSFs can be produced by several microorganisms, including bacteria, and can be used for different purposes [2]. These include the increase of bioavailability of surface-bound and hydrophobic substrates, such as petroleum hydrocarbons (PHs), via direct interfacial contact and pseudo-solubilization [3,4]. BSF production is often associated with the capacity to use hydrocarbons as carbon sources [5] and this combination of traits is particularly advantageous for PHs bioremediation strategies or microbial enhanced oil recovery (MEOR) [3,6]. Since in many cases the contaminated sites and the oil wells are anaerobic environments, anaerobic BSF producing bacteria would be of particular interest. Anaerobic BSF production may also be an advantage at industrial level due to decreased foam formation [7]. However, to date, few microorganisms have been identified as capable of producing BSF in anaerobiosis, with most of these belonging to the *Bacillus* and *Pseudomonas* genera [8].

Hydrocarbonoclastic bacteria are characterized as being able to metabolize one or more PHs by using them as carbon and energy sources, usually preferring these substrates over others [9,10,11]. These bacteria are ubiquitous and can be found both in aerobic and anaerobic environments [12,13,14]. Natural anaerobic biodegradation of PHs occurs primarily in deep subsurface oil reservoirs [15], in the deep ocean near natural seeps [16,17] and in most microaerobic or anaerobic environments contaminated with PHs [18,19,20].

While most of the deep-sea is characterized by low energy, productivity and biological activity rates [21], metabolic activity and biomass is higher in locations where seepage of PHs and other compounds occurs from within deep sediment layers to the seabed, such as hydrothermal vents and cold seeps [22,23]. Cold seeps, including mud volcanoes (MVs), are geologically diverse ecosystems associated with emissions of methane, other PHs and, often, reduced sulfate from the subsurface [24]. Deep-sea sediments, which are often anoxic below a thin upper layer [25], are known sinks for aliphatic and aromatic hydrocarbons [26,27]. Furthermore, bacterial communities from deep-sea surface sediments, in which PHs are present, are enriched in functional genes associated with aerobic and anaerobic degradation of hydrocarbons, in comparison with adjacent sediments without PHs [28,29]. This indicates that deep-sea bacterial communities, particularly in areas where natural seepage occurs, are adapted to PH availability [30]. As such, the deep-sea can potentially be a hidden reservoir of bacterial diversity and novel metabolic activity, with many new bacteria still being discovered [31].

Estuarine systems are sinks for particulate organic matter and contaminants, with PHs being among the most common [32,33]. Sediment deposition and bioturbation can contribute to trap PHs in microaerobic or anaerobic sediment layers [34] in which the presence of PHs further enhances the lowering of the redox potential in sediments [35]. So the presence of PH in estuarine sediments is often associated with microaerobic or anaerobic conditions [36] and estuarine sediments are rich in hydrocarbon-degrading bacteria, especially in contaminated areas [37,38,39].

Due to the difficult access to submerged sediments and the material and time constraints involved in anaerobic cultivation of bacteria, isolation and characterization of culturable anaerobic bacteria from these sediments if often overlooked. The aim of this work is to isolate and identify bacteria with PH-remediation potential from sub-seafloor sediments, in particular from deep-sea and estuarine sites containing PHs. A second objective is to identify potential players in anaerobic natural hydrocarbon biodegradation in marine ecosystems. As such, anaerobic selective cultures were prepared with only crude oil as carbon source and sub-seafloor sediments as inoculum. Bacteria isolated from these cultures were subsequently identified and characterized in terms of BSF production under aerobic and anaerobic conditions.

## 2. Materials and Methods

### 2.1. Sediment Sampling

Estuarine sediments were collected at an unvegetated intertidal site (40°37′32.18″ N, 8°44′09.12″ W) of the estuarine system Ria de Aveiro (NW Portugal). The sampling site is located in the vicinity of the commercial port of Aveiro and other small leisure and artisanal fishing harbors. This area has been previously characterized as contaminated with PHs, both aliphatic and aromatic, and at this particular site surface sediments present contents of 5.81% organic matter, 36.61% moisture and 81.99% fines and mud sediment texture [40]. Sediment samples were collected with a cylindrical steel corer, with 8 cm diameter and 55.5 cm length. Three replicates were collected within an area of 1 m^2^. Each sediment column was composed of an upper layer (~5 cm) of fine sand sediment, an intermediate layer (~10–15 cm) of dark muddy sediments (M) and a bottom medium-grained sand layer (S). Sediments were transported to the laboratory in separated sealed plastic bags and were kept at 4 °C until separation of the layers M and S for further analysis. Corresponding S and M strata of replicate cores were pooled together to obtain composite samples.

Deep-sea sediments were collected during the SWIMGLO/Transflux M86/5 cruise onboard the RV Meteor [41] at the Mikahail Ivanov MV located on the Gulf of Cadiz (Western Mediterranean), which is composed of several craters. Samples were collected at the northwest crater, station 348, which presents no indication of seepage activity, and at the active southeast crater, station 329 (Table 1). Sampling was conducted with a box corer [41]. Sub-samples of 0.5–1 g of subsurface sediments (10 cm below seafloor) were collected, immediately deep-frozen (−80 °C), kept on dry ice during shipping and stored at –80 °C until further analysis.

### 2.2. Anaerobic Selective Cultures—General Procedure

**Figure 1:**
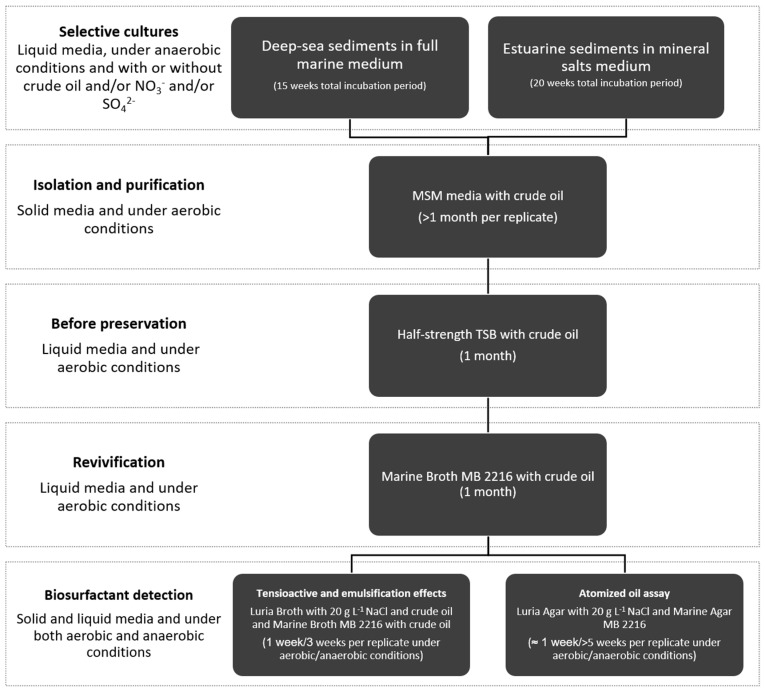
Workflow of the experimental setup for isolation of facultative anaerobic hydrocarbonoclastic biosurfactant-producing bacteria isolated from subaquatic sediments. All steps were carried out at 25 °C.

The general procedure for preparation of anaerobic selective cultures involved the use of a minimum mineral medium, which was different for estuarine or deep marine sediments, to which light Arabian crude oil was added as sole carbon source (Figure 1). Anoxic minimum mineral media [42,43] were amended with resazurin as redox indicator and cycloheximide as anti-fungal agent. All reagents were purchased from Sigma-Aldrich except when otherwise indicated. Aliquots of crude oil were UV-sterilized for four hours, added to culture vials and autoclaved. Unless otherwise mentioned, selective cultures and sub-cultures were prepared in 50 mL serum vials sealed with butyl rubber stops and aluminum crimps. As a final step, after inoculum addition and sealing of the vials, the headspace of the estuary sediment cultures was replaced by a gas mixture of N_2_:O_2_ (85:15) and N_2_ in the deep-sea selective cultures. Vials were kept in inverted position during incubation in the dark at 25 °C [44]. Abiotic controls for all conditions were also included. The detailed procedures are described in the following sections.

#### 2.2.1. Selective Cultures of Estuarine Sediments

A mineral salts medium (MSM) containing NaCl 17.0 g L^−1^, MgCl_2_·6H_2_O 5.8 g L^−1^, Na_2_HPO_4_ 3.0 g L^−1^, NaHCO_3_ 2.5 g L^−1^, KH_2_PO_4_ 2.0 g L^−1^, NH_4_Cl 0.7 g L^−1^, KCl 0.7 g L^−1^, cycloheximide 0.1 g L^−1^, resazurin 1.0 mg L^−1^, FeCl_3_·6H_2_O 0.5 mg L^−1^, ZnCl_2_ 5.0 × 10^−2^ mg L^−1^, CaCl_2_ 2.0 × 10^−2^ mg L^−1^, CuSO_4_ 5.0 × 10^−3^ mg L^−1^ and MnCl_2_·4H_2_O 5.0 × 10^−3^ mg L^−1^ was prepared, and the pH was adjusted to 7.2 ± 0.2 [38]. To select metabolically diverse hydrocarbonoclastic anaerobic bacteria, different terminal electron acceptors and chemical reducing agents (Table 2) [42,43] were used in the selective cultures with different sediment types (Table 3). Flasks were filled with medium to 4:5 of the total volume for fermentative conditions and 9:10 for sulfate reducing bacteria (SRB) and nitrate reducing bacteria (NRB) conditions. Initial cultures were inoculated with 10% (w/v) of the corresponding sediments and 1% (v/v) of crude oil was added to 200 mL flasks. The cultures were incubated for five weeks after which an aliquot of 10% (v/v) was transferred to fresh medium in 50 mL vials. Three more transfers to fresh medium were conducted in the same way so that overall incubation period of the selective cultures was 20 weeks.

#### 2.2.2. Selective Cultures of Deep-Sea Sediments

For the preparation of selective cultures of hydrocarbonoclastic SRB and NRB from deep-sea MVs sediments, an adaptation of the full marine medium [44] was prepared according to Table 4. Resazurin (1 mg L^−1^) was added to the main solution, the vitamin mixture and thiamine supplement were replaced by 10 mL L^−1^ RPMI 1640 Vitamins Solution 100X (Sigma), KNO_3_ was used instead of NaNO_3_ and cycloheximide 0.01 g L^−1^ was added to the final medium.

With the aim of assessing the selective effects of nitrate, sulfate and crude oil amendment on bacterial communities of active and inactive MVs, a factorial experimental design was used. The experimental layout involved 56 serum vials per incubation period, including three sediment sub-samples (identified as a, b and c) from the same corer and abiotic controls (Table 5).

Serum vials were filled up to 9:10 capacity with medium prepared according to the selective factors. In cultures with light Arabian crude oil (C and NC), 1% (v/v) of the carbon source was added and, in cultures without amendment or with only crude (0 and C), KNO_3_ was absent. For the initial cultures, aliquots of 50 mg of sediment were added to 45 mL culture medium, and in the two subsequent transfers to fresh medium, 10% (v/v) of the previous culture was used as inoculum. Incubation in fresh media lasted five weeks, totaling an incubation period of 15 weeks.

### 2.3. Isolation, Purification and Identification of Bacterial Strains

After each incubation period of five weeks, aliquots of 200 µL of each culture were spread-plated on solid MSM medium on which a superficial layer of 50 µL of crude oil had been previously spread. The plates were incubated under aerobic conditions, in the dark, at approximately 25 °C for at least, one month. Randomly selected isolates were purified by consecutively streak-plating three times in identical medium and incubated in similar conditions. Isolates were then inoculated in test tubes with 10 mL half-strength Tryptic Soy Broth (TSB, Liofilchem) and 1 mL crude oil. Aliquots of 1.5 mL of each liquid culture were frozen with glycerol (20%; AppliChem) and kept at −80 °C until processing.

For revivification, aliquots of glycerol-amended frozen cultures were inoculated in 10 mL of Marine Broth MB 2216 (Difco) amended with 100 µL crude oil. The cultures were incubated for one month under aerobic conditions at 25 °C. DNA extraction was performed [45] and the final DNA–RNA pellet was resuspended in 50 µL of TE buffer and stored at −80 °C. BOX-PCR was conducted to identify isolates with similar genotypes, following which clone representatives were selected for identification, thus reducing the DNA sequencing effort. The composition of the reaction mixture (25 μL) was 1 μL of sample, 12.5 μL DreamTaq™ PCR Master Mix (Thermo Fisher Scientific), 0.50 μL of primer, 1.25 μL dimethyl sulfoxide and 9.75 μL dH_2_O. The PCR cycle was 7 min at 94 °C, followed by 35 thermal cycles of 1 min at 94 °C, 2 min at 53 °C and 8 min at 65 °C. A final extension step at 72 °C for 16 min was performed. The primer used was the BOX A1R [46]. PCR products were stored at −20 °C until analysis by gel electrophoresis in agarose gel (1.5%) containing 5.3 × 10^−3^% (v/v) RedSafe, immersed in TAE buffer 1x, at 80 V for 3h. The profiles were visualized using a ChemiDoc XRS+ System scanner and Image Lab software (BioRad, Hercules, CA, USA). The gels obtained were analyzed with BioNumerics v6.6 (Applied Maths, Sint-Martens-Latem, Belgium) and Primer5 software (PRIMER-e, Albany, New Zealand).

The 16S rRNA gene of each BSF-producing bacterial isolate presenting distinct BOX A1R profiles was PCR amplified using the universal bacterial primers 27F and 1492R [47]. The composition of the reaction mixture (25 μL) was 1 μL of sample, 12.5 μL DreamTaq™ PCR Master Mix, 0.25 μL of each primer, 1 μL BSA (2 g L^−1^; Sigma) and 10 μL dH_2_O. The PCR cycle was as described by Domingues et al. [38]. The success of the amplification of the 16S rRNA gene fragments was verified by agarose gel (1%) electrophoresis, with 5.3 × 10^−3^% (v/v) RedSafe (Intron Biotechnology, Gyeonggi-do, South Korea) as DNA staining agent. The amplicons were sequenced by StabVida (Caparica, Portugal). In order to determine their closest relative, the obtained sequences were matched to the sequences available in the GenBank database using BLAST (Basic Local Alignment Search Tool; Bethesda, MD, USA; https://blast.ncbi.nlm.nih.gov/). Sequences can be downloaded from the NCBI (PopSet: 1216628483; Bethesda, MD, USA).

### 2.4. Analysis of Biosurfactant Production

To focus identification efforts on bacteria with biotechnological potential, the ability of isolates to produce BSF was assessed under aerobic conditions using the atomized oil assay [48]. After identification, the isolates were tested for BSF production by the atomized oil assay under both aerobic and anaerobic conditions. After inoculation in LB Agar (Liofilchem) and in Marine Agarose (Marine Broth 2216 supplemented with 2% agarose) with a sterile needle, cultures were incubated under aerobic conditions at 25 °C until small colonies were visible. Identical cultures were incubated in an anaerobic jar, with the gas generator Anaerocult^®^ A (Merck) and the indicator Anaerotest^®^ (Merck), at 25 °C in the dark for a minimum of five weeks. Spraying of the plates with liquid paraffin using an airbrush (model BD-128P, Fengda, China) allowed the detection of halos characteristic of positive results for the production of BSFs. A chemical surfactant solution (25% sodium dodecyl sulfate) and the BSF-producing strain *Pseudomonas* sp. 74 [38] were used as positive controls and *Escherichia coli* DH5α as a negative control.

Tensioactive and emulsification effects were also assessed in cell-free extracts (CFEs). Isolates were cultivated in the media where faster growth was observed by increased turbidity, either LB (Liofilchem) with 20 g L^−1^ NaCl or MB2216, both with 1% crude oil. After one week incubation in aerobic conditions at 25 °C, in the dark, with orbital shaking (100 rpm), 1 mL aliquots were transferred to serum vials with fresh medium containing sodium ascorbate (1.5 mmol L^−1^) as an oxygen scavenger [44]. Anaerobic cultures were incubated for three weeks, at 25 °C, in the dark, with orbital shaking of 100 rpm. In order to prepare CFEs, 50 mL of aerobic and anaerobic cultures were centrifuged at 16,000 g for 5 min and the supernatant was filtered using 0.2 µm pore size PTFE membranes to remove bacterial cells [49].

Surface tension measurements of the CFEs were performed by a surface tensiometer DST9005 (Nima Technology) with a DuNuöy ring at room temperature [50,51]. The E_24_ (emulsification index) was measured after 24 h from emulsion formation as described by Pereira et al. [52] using liquid paraffin (Merck) instead of n-hexadecane. Non-inoculated culture media were used as negative controls in both tests.

All tests were performed in triplicate. Statistical analysis was performed in GraphPad Prism 6 (Graphpad Software Inc, San Diego, CA, USA). BSF production results obtained through the E_24_ and surface tension measurements were compared with the corresponding negative controls by an unvaried analysis of variance (ANOVA) model with the Bonferroni post hoc test. A value of *p* < 0.05 was considered significant.

## 3. Results and Discussion

### 3.1. Isolation and Identification of Isolates

Aiming at selecting hydrocarbonoclastic SRB, NRB or fermentative bacteria from estuarine and deep-sea sediments, anaerobic selective cultures were prepared using only crude oil as carbon source. Thirty-seven hydrocarbonoclastic isolates were retrieved from selective cultures of estuarine sediments and 23 from the selective cultures of deep-sea sediments. After molecular typing, 24 isolates from the estuarine sediments and 11 isolates from the deep-sea sediments were selected for further identification and characterization. Most (14) of the estuarine isolates were obtained from the NRB-selective cultures. An equal number (5) of representative isolates was retrieved from either fermentative or sulfate-reducing cultures. The sediment texture did not appear to affect the isolation of bacteria from the estuarine sediments. On the other hand, in cultures of deep-sea sediments, nearly all isolates, except for DS27, were retrieved from cultures of sediment from the inactive MV. It is possible that the lack of easily accessible carbon sources in the inactive MV, unlike the conditions at active MVs where hydrocarbons are expelled to the hydrosphere, has led to the development of bacteria with alternative strategies to use less available and accessible carbon sources. Overall, all selective cultures were represented in the set of bacterial isolates.

Six isolates from estuarine sediments and an equal number from deep-sea sediments were able to produce BSFs under aerobic conditions as demonstrated by the atomized oil assay. This confirms previous reports on the estuarine system of Ria de Aveiro [38,53] and the deep-sea [27,54] as sources for hydrocarbonoclastic and BSF-producing bacteria. These isolates were identified by sequencing of 16S rRNA gene fragments (Table 6). The most common genera were *Pseudomonas* (DS27, DS192, R47 and R53) and *Ochrobactrum* (R98 and R114). All 12 isolates described here are available upon request addressed to the corresponding author.

Aerobic and anaerobic BSF production are well documented in *Pseudomonas*, including in PH-degrading species [8]. Members of *Ochrobactrum* are known as hydrocarbonoclastic BSF-producers able to degrade hydrocarbons, mainly polycyclic aromatic hydrocarbons (PAHs) [55,56]. They are also known to reduce nitrate [57] or operate fermentative pathways [58] under anaerobic conditions. However, anaerobic production of BSF by *Ochrobactrum* has not yet been reported. Genera *Bacillus*, *Brevundimonas*, *Psychrobacter*, *Staphylococcus*, *Marinobacter* and *Curtobacterium* were also represented (Table 6). All these genera include facultative anaerobes [59,60,61]. *Bacillus* includes some of the most studied BSF-producers under either aerobic or anaerobic conditions, some of them able to degrade PHs [62]. Members of *Curtobacterium* and *Brevundimonas*, in particular, are known for versatile anaerobic metabolism, using several terminal electron acceptors such as nitrate, iron or arsenic [63,64]. *Brevundimonas*, *Staphylococcus*, *Psychrobacter* and *Marinobacter* all include PHs degrading species. The first two are often associated with PAH degradation [65,66], *Psychrobacter* is known to degrade alkanes [67] and *Marinobacter* has been reported to degrade both types of PHs [61]. BSF production under aerobic conditions was also observed in these four genera [68,69,70,71]. However, anaerobic BSF production had not been reported for the first three (*Brevundimonas*, *Staphylococcus* and *Psychrobacter*). A *Marinobacter* member was associated with anaerobic BSF production in a consortium but it was not clear if it was directly responsible for the production [72]. *Curtobacterium* were already isolated from PHs contaminated sediments [73], but this genus has not yet been directly associated with aerobic or anaerobic BSF production or PH degradation. *Pseudomonas* and *Ochrobactrum* were detected in previous studies on PH degrading bacteria populations from the estuary system of Ria de Aveiro [38,74]. While in this study only two members of *Pseudomonas* were isolated from estuarine sediments, it is possible that the anaerobic selective cultures pressured the sediments communities to adapt to the lack of oxygen favoring the growth of facultative anaerobes instead of obligate aerobes. This may also explain the isolation of members of *Marinobacter* and *Curtobacterium*. The former had already been isolated from active salt pans in Ria de Aveiro [75], but the later had not yet been reported in this estuarine system.

All deep-sea isolates identified in this study are represented in the classification of operational taxonomic units from pyrosequencing results [76] and corresponded to genera that were previously reported in marine sediments [55,70,77,78,79,80,81]. The fact that isolates retrieved from selective cultures of MV sediments are associated with the degradation of PHs is consistent with previous findings, in which communities from inactive MVs presented a preference for degradation of PAHs, instead of simpler PHs [76].

### 3.2. Biosurfactant Production

MSM medium was used for isolation of bacteria and for the purification of isolates. However, since the MSM medium is a complex medium requiring laborious preparation, Luria Agar (LA) and Marine Broth 2216 (MB2216), both commercially available, were used to screen for BSF production by the atomized oil assay. LA was successfully used in the cultivation of BSF-producing bacteria previously isolated from the Ria the Aveiro estuary [38] and is also used in the description of the atomized oil assay method [48]. MB2216 was expected to meet the nutritional demands of the isolates as it was designed to mimic the chemical composition of marine environments. As such, it was used as the base for the solid medium with added 2% agarose. NaCl concentration of LA was adjusted to 20 g L^−1^ to match the salinity of MB2216.

All estuarine isolates were able to grow in marine agarose as well as in LA. However, deep-sea isolates DS27 and DS72 only grew in solid MB2216. Other deep-sea isolates, DS140 and DS61, presented faster growth (data not shown) in solid MB2216. Since MB2216 medium is more complex in terms of chemical composition and more specific for marine bacteria, it is possible that it contains some trace nutrients fundamental for the growth of deep-sea bacteria that are lacking in LA. In further tests, LA medium was used to test BSF aerobic and anaerobic production ability in all the estuarine isolates (Table 7) and also for two deep-sea isolates (DS192 and DS104). MB2216 was used for all other deep-sea isolates. For the atomized oil assay both media were used.

**Figure 2:**
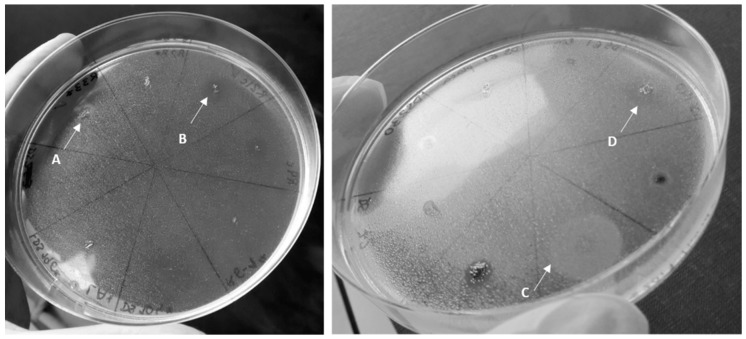
Photographs depicting examples of results of the atomized oil assay for plates incubated in the anaerobic jar. On the left, an LA plate with a bright halo for isolate R33 (A) and a dark halo for R21 (B), both positive results. On the right, a MB2216 plate with a bright halo for the positive control of SDS 25% (C) and a dark halo for DS140 (D).

BSF production was detected in all aerobic and almost all anaerobic cultures using the atomized oil assay (Table 7; Figure 2). The exceptions in anaerobic conditions were isolate DS27, which did not produce BSF in either media, and isolates DS72 and R98, which only produced BSF in LA and MB2216, respectively.

Decrease of surface tension is an effect of the presence of BSF in solutions and is related to its concentration until the critical micelle concentration is reached [82]. Tensiometric measurements indicate that CFEs of aerobic cultures of isolates R33, R47, R53, R98 and R114 caused significant reduction of the surface tension in comparison to the abiotic controls. Significant surface tension decrease was observed under anaerobic conditions only for isolate R47. Since all isolates tested positive for BSF production with the atomized oil assay, it is possible that discrepancies in results obtained with different approaches are related to the sensitivity of the methods. The atomized oil assay is known to be much more sensitive than other methods [48] and is possible that in most cases BSF concentration in CFE was not high enough to present significant decreases in surface tension. In future studies BSFs should be extracted for further characterization, including determination of critical micelle concentration, in order to determine the lowest possible decrease of surface tension.

The E_24_ identifies the presence of emulsifiers, which is often, although not always, a property of BSFs. As such, the results of this method must be interpreted within the framework of other evidence of BSF production, such as provided by the atomized oil assay or surface tension measurements. The E_24_ indicates the presence of molecules that promote stable emulsions for 24 h [83]. Significant emulsification was observed in aerobic cultures of R33, R47, R53 and DS104, and in anaerobic cultures of R53. All significant emulsification effects were obtained with CFE of Luria Broth (LB) cultures. Both LB and MB2216 are very similar in macronutrient composition but it is possible that some of the mineral salts of MB2216 affected BSF structure leading to a decrease in its emulsifying ability. These results also highlight the necessity of using commentary methods to detect bacterial BSF production, since the usage of a single method may lead to false negatives.

#### 3.2.1. Effect of Oxygen Availability

Overall, BSF production was more evident under aerobic conditions in relation to anaerobiosis. In most isolates, CFE of aerobic cultures caused a greater reduction in surface tension (e.g., R47). Higher BSF production in aerobiosis was expected since anaerobic metabolism is slower and energetically less favorable than aerobic metabolism [84]. BSF production by the isolate DS27 was only observed under aerobic conditions, as an exception to all other isolates for which evidence of BSF production could be provided for aerobic and anaerobic cultures. On the other hand, the percentage of BSFs isoforms produced can be affected by the oxygen concentration during growth, leading to the production of BSFs with different characteristics by the same bacteria [85,86]. Further testing is required to characterize the BSFs produced under both aerobic and anaerobic conditions, since a particular structure may have more desirable properties than another that is produced with higher yield.

#### 3.2.2. Effect of Culture Medium in Biosurfactant Production

Optimization of culture media may improve anaerobic BSF production and minimize the effect of oxygen limitation on BSF production yields [87]. In some cases, the same isolates grown on different media produced different sized halos in the atomized oil assay under the same oxygen conditions (e.g., R47 in aerobic conditions; Table 7). Halo size is known to be proportional to BSF concentration [48]. Thus, it is likely that the composition of the culture medium affected BSF production.

When tested by the atomized oil assay, all isolates produced larger or similar-sized halos in LA medium in comparison to MB2216. Considering that LA was adjusted to a similar NaCl concentration than MB2216 (20 g L^−1^), and both media have peptone and yeast extract as carbon sources, the fact that some bacteria prefer LA can possibly be attributed to an inhibiting influence by some of the marine salts present on MB2216. Differences in the type and amount of peptone available in each media my also account for some of the differences in the results. Furthermore, under anaerobic conditions, BSF production by DS72 was only detected in LA, while by R98 was only observed in MB2216. Since MB2216 is a more complex media than LA, it is possible that some nutrients present in the former are essential to BSF production by some bacteria. As such, the choice of medium used in the atomized oil assay could, in the extreme, lead to false negatives, especially considering that LA is a medium used in the original description of the method [48].

#### 3.2.3. Biosurfactant Production in Estuarine and Deep-Sea Bacterial Isolates: Biotechnological Potential

Overall, the most promising isolates for the production of BSF in aerobic conditions were retrieved from selective cultures of estuarine sediments (Table 7). In this set of isolates, R53 was the only one with significant emulsification in aerobic and anaerobic CFEs, indicating the production of a BSF with a strong emulsifying effect. Under aerobic conditions, CFEs of R114 and R98 were also associated with significant decreases of surface tension and both produced large halos (>0.5 cm) in the atomized oil assay. R47 was a promising isolate for both aerobic and anaerobic BSF production since it caused the greatest decrease of surface tension both in aerobic and anaerobic cultures, as well as the highest E_24_ and the second largest halo for aerobic cultures.

Some of the most promising isolates from the deep-sea were DS61 and DS140, which produced the largest halos in anaerobic conditions. This may indicate that bacteria from these environments are probably more adapted to the production of BSF under oxygen limiting conditions. DS104 was the only deep-sea isolate with significant E_24_ determined for aerobic cultures.

Some of the most promising BSF producers found in this study are included in genera for which BSF production had not yet been reported. For isolate R33, identified as a *Curtobacterium* sp., BSF production was for the first time detected in aerobic and in anaerobic cultures. BSF production under oxygen limitation by isolates belonging to *Ochrobactrum* sp. (R98 and R114), *Psychrobacter* sp. (DS104) and *Staphylococcus* sp. (DS140) was also observed for the first time. This is also the case of the less productive isolates identified as *Brevundimonas* and *Marinobacter*, DS72 and R21, respectively. This is probably due to the screening for BSF production in isolates from environmental matrices being frequently conducted only in aerobic conditions due to material and time constrains [8].

*Pseudomonas* sp. 74, a BSF-producing bacterium previously isolated from the rhizosphere of halophytes in the estuary of Ria de Aveiro [38], was used as positive control of the aerobic atomized oil assay in solid MB2216 and in LA medium. This isolate was also tested in anaerobic conditions and BSF production was confirmed. As such, it is also a promising candidate for further studies to detail anaerobic BSF production.

Due to their ability to use PHs as carbon sources and produce BSFs in the absence of oxygen, the isolates described in this work could represent strains with biotechnological potential for industrial BSF production, bioremediation for decontamination of petroleum in marine sediments and MEOR strategies. They could be of particular interest for applications requiring anaerobic conditions or, as aerotolerants, in environments that oscillate between aerobiosis and anaerobiosis. Furthermore, the bacteria now identified are halotolerant, making them good candidates for biotechnological applications requiring BSF production in high salinity levels. At industrial level, the high salinity levels required for BSF production by the isolates may represent an advantage since the survival of contaminants is so low that sterility requirements may be looser to lower production costs. However, for some isolates, particularly for those from the deep-sea, bacterial growth is slow, possibly because culturing conditions were very different from the ones in their habitats. Future work should involve the characterization of the produced BSFs and the optimization of BSF production.

## 4. Conclusions

Twelve isolates belonging to *Pseudomonas*, *Bacillus*, *Ochrobactrum*, *Brevundimonas*, *Psychrobacter*, *Staphylococcus*, *Marinobacter* and *Curtobacterium* genera were isolated from selective cultures prepared with deep-sea and estuarine subaquatic sediments. From these the first two are well described as anaerobic BSF producers. However, for *Ochrobactrum*, *Brevundimonas*, *Psychrobacter*, *Staphylococcus*, *Marinobacter* and *Curtobacterium*, to the best of the authors’ knowledge, this was the first time that members of these genera were linked to anaerobic production of BSFs. All isolates were able to grow with crude oil as sole carbon source and all produced BSF under aerobic and anaerobic conditions, with the exception of DS27 which only did so in aerobic conditions. Subaquatic sediments of marine environments were confirmed to represent a valuable seedbank for the isolation of aerobic and anaerobic hydrocarbonoclastic and BSF-producing bacteria. More importantly, the still fairly unknown deep-sea sediments may provide new strains with biotechnological potential and, consequently, open perspectives for novel BSFs.

## Figures and Tables

**Table 1:** Description of sampling stations at the Mikahail Ivanov mud volcano.

Station	Latitude (D:M)	Longitude (W)	Depth (m)	Activity
329	35:44.34	10:12.06	4492	Active
348	35:44.41	10:12.18	4497	Inactive

**Table 2:** Selective supplements to mineral salts medium (MSM).

Supplements	Fermentative	SRB	NRB	Solid MSM
0.5 M Na_2_SO_4_ (mL L^−1^)	-	28	-	28
0.5 M KNO_3_ (mL L^−1^)	-	-	40	40
0.2 M Na_2_S 9H_2_O (mL L^−1^)	5	5	-	-
0.2 M Cysteine-HCl (mL L^−1^)	15	15	-	-
0.5 M Ascorbic acid (mL L^−1^)	-	-	8	8
Agarose (g L^−1^)	-	-	-	20
Crude oil (% v/v)	1	1	1	50 µL ^a^

^a^ Per plate, spread on the surface of the culture medium.

**Table 3:** Selective cultures of estuarine sediments in relation to sediment type and selective pressure (in italics) for certain anaerobic metabolic types. NRB—nitrate reducing bacteria; SRB—sulfate reducing bacteria; Fer—fermentative bacteria.

		Selective Pressure		
		*NRB*	*SRB*	*Fermentative*
**Inocula**	*Abiotic control*	Ø -NRB	Ø -SRB	Ø -Fer
	*Mud*	M-NRB	M-SRB	M-Fer
	*Sand*	S-NRB	S-SRB	S-Fer

**Table 4:** Supplements used in cultures prepared with deep-sea sediments according to the different selective factors (in italics).

	Selective Pressure (Added Electron Acceptors)							
	*Without Crude*				*With Crude*			
	*None*	*NO_3_^−^*	*SO_4_^−2^*	*NO_3_^−^ + SO_4_^−2^*	*None*	*NO_3_^−^*	*SO_4_^−2^*	*NO_3_^−^ + SO_4_^−2^*
**Na_2_SO_4_ (g L^−1^)**	0.5 ^a^	0.5 ^a^	4.0	4.0	0.5 ^a^	0.5 ^a^	4.0	4.0
**KNO_3_ (g L^−1^)**	-	1.0	-	1.0	-	1.0	-	1.0
**Reducing agent** **Na_2_S · 9H_2_O (g L^−1^)**	-	-	0.24	0.24	-	-	0.24	0.24
**Inoculum (mL L^−1^)**	100	100	100	100	100	100	100	100
**Crude oil (mL L^−1^)**	-	-	-	-	44.4	44.4	44.4	44.4

^a^ Present in basal medium.

**Table 5:** Identification of selective cultures of deep-sea mud volcano sediments amended with different electron acceptors (in italics). Whenever sub-sample identification is required the letters a, b and c are used for the different sub-samples.

		Selective Pressure							
		*Without Crude*				*With Crude*			
		*None*	*NO_3_^−^*	*SO_4_^−2^*	*NO_3_^−^ + SO_4_^−2^*	*None*	*NO_3_^−^*	*SO_4_^−2^*	*NO_3_^−^ + SO_4_^−2^*
**Inocula**	*Abiotic control*	Ø-0	Ø-N	Ø-S	Ø-NS	Ø-C	Ø-NC	Ø-SC	Ø-NSC
	*Active MV*	A-0	A-N	A-S	A-NS	A-C	A-NC	A-SC	A-NSC
	*Inactive MV*	I-0	I-N	I-S	I-NS	I-C	I-NC	I-SC	I-NSC

**Table 6:** BLAST results obtained for the 16S rRNA gene sequence alignments of the 12 biosurfactant-producing isolates. DS indicates isolates from the deep-sea selective cultures and R from the estuarine selective cultures.

Isolate No.	Selective Culture	Sequence Accession No.	Closest Relative		
			BLAST-N Identity	Accession No.	% Identity
**DS27**	Ab-0	MF490026	*Pseudomonas sp.*	KR088605	99
**DS61**	Ia-N	MF490027	*Bacillus subtilis*	KU862331	100
**DS72**	Ic-S	MF490028	*Brevundimonas sp.*	LN833256	100
**DS104**	Ib-NS	MF490029	*Psychrobacter sp.*	KF859544	100
**DS140**	Ic-C	MF490030	*Staphylococcus sp.*	KT282233	99
**DS192**	Ic-NC	MF490031	*Pseudomonas sp.*	KX301316	100
**R21**	S-Met	MF490033	*Marinobacter salsuginis*	KM041132	99
**R33**	S-SRB	MF490034	*Curtobacterium flaccumfaciens*	KF003415	100
**R47**	M-SRB	MF490035	*Pseudomonas xanthomarina*	KY465430	99
**R53**	M-NRB	MF490036	*Pseudomonas sp.*	LN877946	99
**R98**	M-NRB	MF490037	*Ochrobactrum sp.*	KJ676969	100
**R114**	S-SRB	MF490038	*Ochrobactrum sp.*	JQ014582	100

**Table 7:** Characterization of biosurfactant production indicators by identified isolates.

Isolates	Atomized Oil Assay (Halo Diameter in cm)				Surface Tension Reduction * (mN/m)		Emulsification Index E_24_ (%)	
	*LA* *Aer.*	*LA* *Anaer.*	*MA* *Aer.*	*MA* *Anaer.*	*Aer.*	*Anaer.*	*Aer.*	*Anaer.*
DS27	0.6 ± 0.21 ^a^	0.0 ± 0.00 ^b^	0.4 ± 0.21 ^a^	0.0 ± 0.00 ^b^	3.3 ± 2.51	1.4 ± 0.97	2.5 ± 0.14	0.0 ± 0.00
DS61	0.6 ± 0.16 ^a^	0.3 ± 0.07 ^a^	0.6 ± 0.15 ^a^	0.8 ± 0.39 ^a^	2.0 ± 0.89	3.9 ± 6.97	3.5 ± 0.19	7.8 ± 6.34
DS72	0.4 ± 0.04 ^a^	0.5 ± 0.44 ^a^	0.4 ± 0.04 ^a,b^	0.0 ± 0.00 ^b^	1.9 ± 1.68	1.6 ± 2.56	0.0 ± 0.00	5.2 ± 4.53
DS104	0.7 ± 0.23 ^a^	0.3 ± 0.04 ^b^	0.5 ± 0.22 ^a,b^	0.5 ± 0.22 ^a,b^	1.9 ± 2.80	1.2 ± 2.08	**60.6 ± 9.32**	7.3 ± 3.43
DS140	0.9 ± 0.31 ^a^	1.3 ± 0.45 ^b^	0.6 ± 0.15 ^a^	1.0 ± 0.49 ^a,b^	−1.4 ± 2.11	−1.7 ± 4.16	7.1 ± 0.85	2.9 ± 0.79
DS192	0.7 ± 0.30 ^a^	0.3 ± 0.16 ^a,b^	0.5 ± 0.17 ^a,b^	0.1 ± 0.04 ^b^	−0.3 ± 0.97	1.5 ± 3.11	8.2 ± 1.21	13.5 ± 4.84
R21	1.3 ± 0.39 ^a^	0.5 ± 0.24 ^b^	0.6 ± 0.24 ^b^	0.2 ± 0.11 ^b^	1.4 ± 2.96	−0.2 ± 3.92	12.3 ± 10.27	6.3 ± 3.84
R33	0.6 ± 0.12 ^a^	0.1 ± 0.10 ^b^	0.6 ± 0.23 ^a^	0.2 ± 0.15 ^b^	**7.1 ± 1.65**	0.6 ± 3.75	**38.6 ± 13.50**	8.0 ± 3.36
R47	1.1 ± 0.40 ^a^	0.5 ± 0.13 ^b^	0.4 ± 0.29 ^b^	0.3 ± 0.11 ^b^	**9.9 ± 0.37**	**4.5 ± 3.57**	**61.2 ± 6.41**	0.0 ± 0.00
R53	0.7 ± 0.09 ^a^	0.5 ± 0.32 ^a^	0.3 ± 0.13 ^a^	0.4 ± 0.13 ^a^	**5.3 ± 1.64**	2.0 ± 0.55	**44.7 ± 5.83**	**35.0 ± 9.31**
R98	0.5 ± 0.16 ^a,c^	0.0 ± 0.00 ^b^	0.8 ± 0.39 ^a^	0.3 ± 0.22 ^b,c^	**6.3 ± 2.76**	2.1 ± 0.64	10.3 ± 6.84	6.7 ±4.33
R114	0.6 ± 0.15 ^a^	0.3 ± 0.22 ^a^	0.7 ± 0.18 ^a^	0.3 ± 0.31 ^a^	**6.7 ± 3.16**	0.7 ± 1.09	13.1 ± 5.27	18.0 ± 11.32
*E. coli* DH5α	0.0 ± 0.00 ^a^	0.0 ± 0.00 ^a^	0.0 ± 0.00 ^a^	0.0 ± 0.00 ^a^	-	-	-	-
*Pseudomonas* sp. 74	1.2 ± 0.35 ^a^	0.8 ± 0.58 ^a,b^	1.0 ± 0.57 ^a,b^	0.7 ± 0.47 ^b^	-	-	-	-

* Surface tension reduction values correspond to the difference between a cell-free extract of each culture and the corresponding abiotic control (culture medium). Numbers in bold correspond to statistically significant values compared to abiotic controls (*p* < 0.05). Different letters in superscript indicate significant differences per isolate across the different media/oxygen conditions (*p* < 0.05). LA—Luria Agar; MA—Marine Agarose 2216; Aer.—Aerobic cultures; Anaer.—Anaerobic cultures.
